# Supplementary material for: Functional Characterization of the N-Acetylmuramyl-l-Alanine Amidase, Ami1, from Mycobacterium abscessus
Source: Cells. 2020 Nov 4;9(11):2410. doi: 10.3390/cells9112410 (PMC7694207; doi:10.3390/cells9112410)
Supplement: Supplementary file 1 [file cells-09-02410-s001.pdf]

cells

Table S1. Primers used in this study

Mab0318_pUX1katG_LA_F	CTAGCTAGCTCCTATGCGGGTCAACAGTTCCCG (*Nhe1*)
Mab0318_pUX1katG_LA_R	GAAAAGGGTTAGCCTCGCACGTCGCCACGGTAG
Mab0318_pUX1katG_RA_F	CGACGTGCGAGGCTAACCCTTTTCCACCACCTGC
Mab0318_pUX1katG_RA_R	CTACAATTGTGGGTGAACCGCGCCC (*MfeI*)
Mab0318_pMV306hsp_EcoRI_F:	CGGAATTCATGCGAGTGAGTTTCTGGCGCGCCGCG (*EcoRI*)
Mab0318_SalI_CtermStrepTag	CGCGTCGACTTACTTCTCGAACTGCGGGTGGCTCCAGCCGCCGGCGGGCGCGGCCTCG (*SalI*)
Mab0318_pET151_F	CACCGCACCCGGTATCGCCGGACGCATCGTTGTGCTCGATCCC
Mab0318_pET151_R	GAGGAGAAGCCCGGTTAGCCGCCGGCGGGCGCGGCCTCGGGCGCC
Smeg6281_pET30_F	CGGGGTACCGAGAATCTGTACTTCCAGGGAGCCCCCTCCAACATCGCCGGAATGATCGTGTTCCTCGATCCC (*KpnI*)
Smeg6281_pET30_R	CCGGAATTCTCAACGCACGGGGCTGACGGCCGCCGCGGCCTGCG (*EcoRI*)
Mtb_3717_F	GACGACGACAAGATGGAGAATCTGTACTTCCAGGGAACCCCCGCCAACATCGCCGGCATGGTCGTCTTCATCGAC
Mtb_3717_R	GAGGAGAAGCCCGGTTAACGCGCCTGGCCCTGGGTGGCCAGGAAGCCGGC

Restriction sites are in italic and indicated between parentheses.

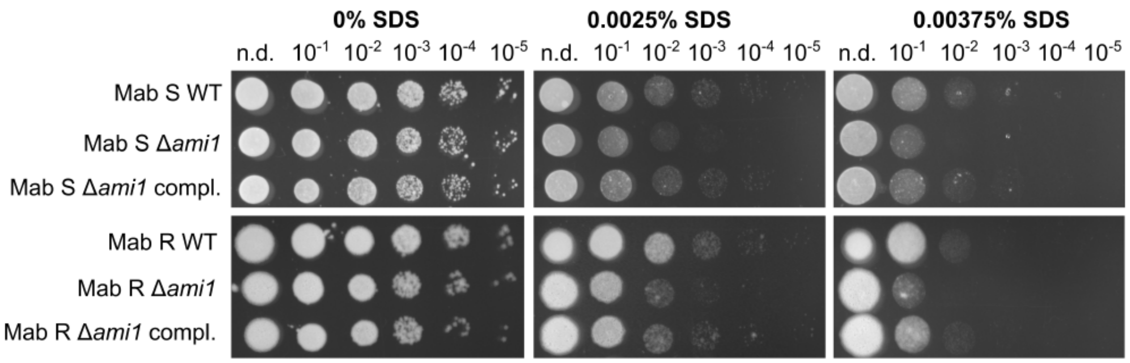

Figure S1. Detergent susceptibility assay. Bacterial cells were serially diluted and spotted on LB agar plates in the absence or presence of SDS at 0.0025 and 0.0037% (*v*/*v*) and incubated at 37 °C for 3 days. Non-diluted cultures in the exponential growth phase were adjusted to an OD of 0.8 and serially diluted 10-fold. n.d. indicates non diluted.
